# Grading pharmacists’ risk of complaints to a regulator: A retrospective cohort study

**DOI:** 10.3389/jpps.2023.11228

**Published:** 2023-03-21

**Authors:** Katherine Morris, Matthew J. Spittal

**Affiliations:** ^1^ Information & Data Management, Ontario College of Pharmacists, Toronto, ON, Canada; ^2^ Melbourne School of Population and Global Health, The University of Melbourne, Carlton, VIC, Australia

**Keywords:** patient complaints, quality and safety, risk prediction, pharmacists, pharmacists regulation

## Abstract

**Background:** Tools to grade risk of complaint to a regulatory board have been developed for physicians but not for other health practitioner groups, including pharmacists. We aimed to develop a score that classified pharmacists into low, medium and high risk categories.

**Methods:** Registration and complaint data were sourced from Ontario College of Pharmacists for January 2009 to December 2019. We undertook recurrent event survival analysis to predict lodgement of a complaint. We identified those variables that were associated with a complaint and included these in a risk score which we called PRONE-Pharm (Predicted Risk of New Event for Pharmacists). We assessed diagnostic accuracy and used this to identify thresholds that defined low, medium and high risk.

**Results:** We identified 3,675 complaints against 17,308 pharmacists. Being male (HR = 1.72), older age (HR range 1.43–1.54), trained internationally (HR = 1.62), ≥1 prior complaint (HR range 2.83–9.60), and complaints about mental health or substance use (HR = 1.91), compliance with conditions (HR = 1.86), fees and servicing (HR = 1.74), interpersonal behaviour or honesty (HR = 1.40), procedures (HR = 1.75) and treatment or communication or other clinical issues (HR = 1.22) were all associated with lodgement of a complaint. When converted into the PRONE-Pharm risk score, pharmacists were assigned between 0 and 98 points with higher scores closely associated with higher probability of a complaint. A score of ≥25 had sufficient accuracy for classifying medium-risk pharmacists (specificity = 87.0%) and ≥45 for high-risk pharmacists (specificity = 98.4%).

**Conclusion:** Distinguishing isolated incidents from persistent problems poses a significant challenge for entities responsible for the regulation of pharmacists and other health practitioners. The diagnostic properties of PRONE-Pharm (minimizing the false positives) means that the risk score is useful for “ruling-out” low risk pharmacists using routinely collected regulatory data. PRONE-Pharm may be useful when used alongside interventions appropriately matched to a pharmacist’s level of risk.

## Background

In the field of medicine, risk scores are increasingly being used to identify physicians at high risk of complaints to medical boards (1, 2). These scores take characteristics known to be associated with complaint risk (for example, male sex, number of prior complaints) and assign points to each predictor based on the level of associated risk. Points are summed to produce a total score. A threshold is then identified that stratifies physician scores into “low risk” and “high risk” categories. Because a small number of physicians routinely accrue the lion’s share of complaints (3, 4), risk scores allow a risk-based regulation approach to be adopted, meaning interventions to remediate performance problems can largely be targeted at the small group of risky physicians.

Little comparable work has been undertaken in relation to pharmacists to identify either the characteristics of those at risk of complaints or to take the next step of aggregating information into a risk score. An early study from the UK found that the incidence of disciplinary action against pharmacists by the Royal Pharmaceutical Society were low (<1% of pharmacists per year) and that failure to keep written records and fraud were the most common reasons for disciplinary action (5). Another UK study compared pharmacists referred to a disciplinary committee to a matched sample of pharmacists not subject to disciplinary proceedings (6). Only one factor, working in a community pharmacy (as opposed to a hospital pharmacy), emerged as a predictor of disciplinary action. A cross-sectional survey in Northern Ireland found that work factors and well-being factors were associated with risk-taking, a behaviour thought to compromise fitness to practice (7). In a recent case-series analysis of disciplinary cases from Canada (8), most events leading to disciplinary action occurred in a community pharmacy and were not caused by isolated errors.

Ideally, the factors associated with complaint risk would be studied longitudinally as this captures the changing nature of risk over time, for instance, as pharmacists accrue additional complaints. This approach accords with how regulatory data is typically captured by boards as they discharge their duties. It also facilitates a more direct translation of any risk score derived from research into a tool that can be used by boards. One Australian study did this (2). Data from 14 health professional groups—including pharmacists—were used to identify the factors associated with complaints to the relevant board about a practitioner, and these results were then translated into a risk score called PRONE-HP (Predicted Risk of a New Event for Health Practitioners). The results showed, however, that the diagnostic accuracy of PRONE-HP was not high enough for pharmacists for the tool to be routinely used in that setting. This may be because complaints against pharmacists are relatively rare in Australia, but more fundamentally, because the aim of that study was to develop a generic risk score that could be used for all 14 practitioner groups. A better approach may be to develop a pharmacist-specific risk score.

The aim of the study was to do develop a risk score specifically for pharmacists. Using data from Ontario, Canada, we sought to undertake a longitudinal study of all registered pharmacists to identify the factors associated with complaint risk. We then sought to convert these findings into a points-based risk score that could be used to classify a pharmacist’s risk level for complaints into three categories: low, medium and high.

## Methods

### Setting

In Canada, pharmacy is a self-regulating profession and each province has their own regulatory body (9). In Ontario, pharmacists are regulated by the Ontario College of Pharmacists (OCP) (10). The Inquiries, Complaints and Reports Committee (ICRC) oversees all complaints and investigations into a professional’s conduct and competence (11). The study was conducted in accordance with the Declaration of Helsinki, and the protocol was approved by the OCP ethics committee (2020-07-OKDA).

## Registration and complaints data

We extracted information from OCP operational databases for all pharmacists registered to practice in Ontario between 1 January 2009 and 31 December 2019. The registration data consisted of information on each pharmacist’s age, gender, years of OCP registration, years since graduating and place of qualifying education. In addition, we accessed data indicating a financial interest in a pharmacy (i.e., whether a pharmacist was a shareholder or director).

We also identified information on all complaints about these pharmacists lodged with OCP during the same time period. The complaints data included the date of complaint and the main issues raised by the complainant. The issue raised in each complaint was recorded by OCP staff at lodgement. We coded these into one of three categories and 12 subcategories used previously (12). These were health issues (mental health or substance use); conduct issues (compliance with conditions, fees and servicing, interpersonal behaviour or honesty, records and reports, sexual boundaries, use or supply of medications, other conduct issues); performance issues (prescribing and dispensing, procedures, treatment or communication or other clinical issues); and unknown issues.

### Study dataset

We built a person-period dataset in which each row of data represented covariate values for a pharmacist for each time interval they were at risk of a complaint. New intervals created new rows of data, which began on the date the value of a time-varying variable changed and ended at the next change of any time-varying variable. The values for a practitioner’s sex (male, female) and place of qualifying education (Canada and US vs. International) did not change over time. All other variables could be time varying. We coded age into nine categories (≤29, 30–34, 35–39, 40–44, 45–49, 50–54, 55–59, 50–64, ≥65), and pharmacists could move from one category to another as they aged. We coded a variable representing the number of prior complaints during the study period (0, 1, 2, 3, 4, ≥5) and a variable for financial interest in a pharmacy (Yes, No). We constructed look-back variables for 12 complaint issues, representing the presence or absence of a complaint about that issue in the past 2 years.

### Statistical analyses

We first calculated the number of complaints and the unadjusted complaint rate (per 1,000 person years) for the sample as a whole and by practitioner and complaint characteristics. Next, we built the complaint risk calculator in three steps outlined below. To develop and validate the risk score, we first randomly split the data into a training sample (70% of pharmacists) and a validation sample (the remaining 30% of pharmacists). Analyses were performed on complete-case records, meaning there was no missing data on any study variables.

### Predicting complaints

We used survival analysis to identify characteristics of practitioners at risk of one or more complaints. We followed our previous approach (2) and used an Anderson-Gill model (13) which allows each pharmacist to accrue multiple complaints over the study period. Cluster-adjusted robust standard errors were calculated to account for multiple periods of observation per practitioner. The predictors were pharmacist age, sex, country of training, number of prior complaints, years of OCP registration, years since graduation, financial interest in a pharmacy and the 12 complaint issues, excluding the unknown category. After fitting an initial model, we excluded non-significant predictors (years of OCP registration, years since graduation, financial interest in a pharmacy) to arrive at a final model. We assessed the fit of this model by applying the log-hazard ratios (the coefficients) developed on the training sample to the validation sample and calculating the C-index.

### Building the risk score (PRONE-Pharm)

We used the results of the final survival model to design a scoring system. Each risk factor was assigned points, where the number of points assigned was scaled directly from the coefficients of the model. Specifically, we multiplied the log hazard ratios for each predictor by 14.9 and then rounded to the nearest whole number. (This value was chosen by taking the inverse of the smallest coefficient in the model and means the variable associated with this coefficient had a score of 1 point.) This scoring model allows for a simple calculation of a pharmacist’s risk score each time a new complaint is lodged against them. We refer to the risk-score as PRONE-Pharm: Predicted Risk of New Event for Pharmacists.

### Evaluating the performance of PRONE-Pharm

We assessed the performance of PRONE-Pharm in two ways. First, to assess calibration of the risk score, we calculated and compared Kaplan-Meier curves for 4 score ranges in the training and validation samples and plotted these. Second, to assess accuracy, we calculated the sensitivity and specificity for the prediction of a new complaint within 2 years for different thresholds. We did this using methods appropriate for censored data (14).

## Results

### Sample characteristics

The data consisted of 17,038 pharmacists registered to practice between January 2009 and December 2019. Fifty-eight percent were female, 94% were under 65 years of age and 59% were educated in Canada and the US (Table 1). These practitioners had a total of 3,675 complaints (mean 1.38 complaints per person). Concerns about prescribing and dispensing were the most frequent reason for complaints (39.0%), followed by treatment or communication or other clinical issues (21.9%) and procedures (15.5%).

**TABLE 1:** Characteristics of pharmacists and complaints.

	N	Percent
Characteristics of pharmacists	17,038	100
Gender		
Female	9,885	58
Male	7,153	42
Age at baseline		
≤29	5,672	33.3
30–34	2,751	16.1
35–39	2,541	14.9
40–44	2,081	12.2
45–49	1,676	9.8
50–54	1,229	7.2
55–59	658	3.9
60–64	284	1.7
65+	146	0.9
Country of training		
Canada and US	10,075	59.1
International	6,963	40.9
Financial interest in pharmacy		
Shareholder or director	5,106	30
No financial interest	11,932	70
Characteristics of complaints	3,675	100
Health: Mental health or substance use	53	1.4
Conduct: Compliance with conditions	30	0.8
Conduct: Fees and servicing	333	9.1
Conduct: Interpersonal behaviour or honesty	507	13.8
Conduct: Records & reports	131	3.6
Conduct: Sexual boundaries	26	0.7
Conduct: Use or supply of medications	71	1.9
Conduct: Other conduct issues	330	9
Performance: Prescribing or dispensing	1,435	39
Performance: Procedures	571	15.5
Performance: Treatment or communication or other clinical issues	806	21.9
Unknown/unclassified issues	141	3.8

### Factors associated with risk of complaint

After excluding non-significant predictors, we identified a set of variables associated with complaint risk (Table 2). Male pharmacists’ complaint risk was 1.72 times that of females after adjustment for all other variables. Compared to pharmacists aged ≥65 years (the age group with the lowest risk and the reference category), the risk of a complaint was lowest for those aged ≤29 years (HR = 1.21) and increased after that (HR range: 1.43–1.54) until ages 60–64 years where it declined slightly (HR = 1.32). Pharmacists trained internationally had a higher risk (HR = 1.62) than those trained in the Canada and the US.

**TABLE 2:** Complaint rates and survival model with risk scores.

Variable	Number of complaints^a^	Rate (per 1000 PY)^a^	Model HR (95% CI)^b^	Risk score^b^
Sex				
Female	1,504	18.7	Ref.	0
Male	2,171	37.6	1.72 (1.58–1.87)	8
Age at baseline				
≤29	265	17.2	1.21 (0.94–1.55)	3
30–34	493	23.9	1.43 (1.14–1.79)	5
35–39	528	26.0	1.45 (1.15–1.81)	5
40–44	583	27.8	1.43 (1.15–1.78)	5
45–49	558	28.7	1.36 (1.09–1.69)	5
50–54	487	30.0	1.50 (1.20–1.90)	6
55–59	377	30.4	1.54 (1.22–1.93)	6
60–64	243	32.4	1.32 (1.03–1.68)	4
65+	141	26.6	Ref.	0
Country of training				
Canada and US	1,702	19.7	Ref.	0
International	1,973	38.0	1.62 (1.49–1.77)	7
Number of prior complaints				
0	2,668	21.1	Ref.	0
1	666	69.6	2.83 (2.52–3.18)	16
2	196	123.0	4.24 (3.48–5.17)	22
3	71	185.8	6.19 (4.52–8.47)	27
4	36	273.9	5.44 (3.12–9.48)	25
≥5	38	431.2	9.60 (5.36–17.2)	34
Complaint issues (all in the last 2 years)				
Health: Mental health or substance use (Ref. = No)	14	176.3	1.91 (1.10–3.34)	10
Conduct: Compliance with conditions (Ref. = No)	18	384.1	1.86 (1.12–3.08)	9
Conduct: Fees and servicing (Ref. = No)	109	224.5	1.74 (1.27–2.37)	8
Conduct: Interpersonal behaviour or honesty (Ref. = No)	108	111.4	1.40 (1.08–1.81)	5
Performance: Procedures (Ref. = No)	137	166.7	1.75 (1.38–2.23)	8
Performance: Treatment or communication or other clinical issues (Ref. = No)	121	116.5	1.22 (0.97–1.53)	3
C-index (95% CI)			0.70 (0.69–0.71)	

^a^ Calculated using the whole sample;

^b^ Calculated using the training sample (randomly selected 70% of pharmacists).

Complaint risk increased with the number of prior complaints. Compared to those with no prior complaints, pharmacists with one prior complaint had 2.83 times higher risk of getting another complaint. Those with three prior complaints had 6.19 times the risk, and those with ≥5 complaints had 9.6 times the risk. The complaint issues most strong related to a subsequent complaint were problems with compliance with conditions (HR = 1.86), mental health or substance use problems (HR = 1.91), problems with procedures (HR = 1.75) and problems with fees and servicing (HR = 1.74). PRONE-Pharm showed good discrimination when applied to the training dataset (C-index = 0.70).

### Performance of PRONE-Pharm

Table 2 (far right column) shows the points assigned to each predictor, based directly on the hazard ratios estimated in the survival model. The largest number of points were for number of prior complaints, with 34 points assigned to a pharmacist with ≥5 prior complaints. The minimum total score is 0, which would be assigned to a female pharmacist, aged ≥65 years, trained in Canada or the US and with no prior complaints. The maximum score is 98, corresponding to being male (8 points), aged 50–54 or 55–59 (6 points), trained internationally (7 points), ≥5 prior complaints (34 points), and complaints in the last 2 years for problems with mental health or substance use (10 points), problems with compliance with conditions (9 points), problems with fees and servicing (8 points), problems with procedures (8 points), problems with interpersonal behaviour or honesty (5 points) and problems with treatment or communication or other clinical issues (3 points).

**FIGURE 1:**
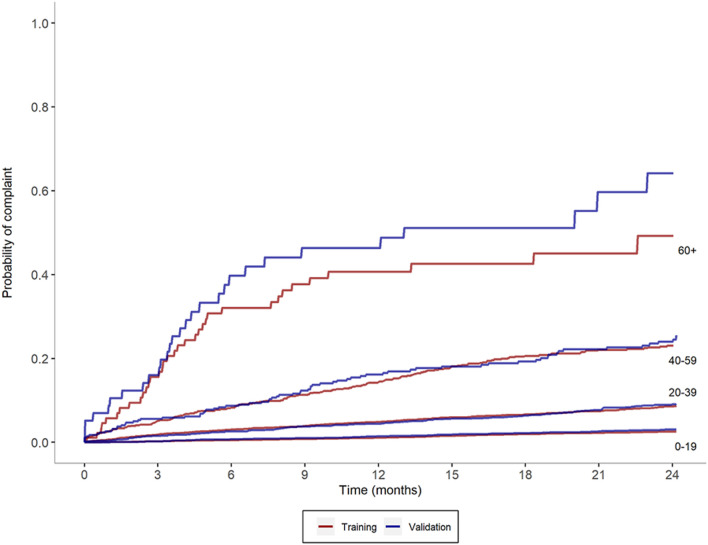
Observed probability of complaints based on selected risk score ranges for test and validation samples.

To assess the calibration of PRONE-Pharm, we examined the out-of-sample consistency within risk strata. Figure 1 shows Kaplan-Meier curves plotting the probability of a subsequent complaint for four selected risk score ranges. Within each range two curves are displayed: one from the training and validation datasets respectively. The pairs of curves in the lowest four ranges show very close concordance. In the highest range (risk score 60 or more) there is some divergence in the pair of curves, particularly after 6 months. This divergence is due to the low number of pharmacists (30) that were placed in that group, however the vast majority of high risk individuals are not in the 60+ group, but rather in the 40–59 group where alignment is very good.

Figure 1 also shows a high degree of consistency up and down the risk scale. The probability of a subsequent complaint increases monotonically across the ranges. For example, at 24 months the probability of a further complaint is 2.6% for pharmacists in the 0–19 point range, 8.9% for pharmacists in the 20–39 point range, 24.3% for pharmacists in the 40–59 point range, 46.5% for pharmacists in the 60+ point range.

### Thresholds for identifying medium-risk and high-risk pharmacists using PRONE-Pharm

Table 3 shows the diagnostic accuracy of PRONE-Pharm for thresholds ranging from a total score of ≥10 to ≥90. Ideally, both sensitivity and specificity would be high, but there is a trade-off between the two such that a high value on one means a low value on the other. We therefore focus on thresholds that maximise specificity because we want to minimise the false positives given the consequence a medium or high-risk classification may have on a pharmacist’s practice. For a medium risk classification, a threshold of ≥25 may be appropriate. At this level, sensitivity is 24.7%, specificity is 87.0% and around 13% of pharmacists would be classified as medium risk or above. For a high-risk classification, a threshold ≥45 may be appropriate. This threshold has sensitivity of 4.7% and specificity of 98.4%. Approximately 1.6% of pharmacists would be classified as high risk according to this criterion.

**TABLE 3:** Diagnostic properties of the risk score: predicting new complaint within 2 years.

Risk category	Threshold	Sensitivity (%)	Specificity (%)	No. Pharmacists	Percent of all pharmacists
Low	≥5	87.2	24.3	15,195	89.2
	≥10	82.6	30.4	10,945	64.2
	≥15	49.9	65.9	5,193	30.5
	≥20	42.9	71.7	4,974	29.2
Medium	≥25	24.7	87.0	2,220	13.0
	≥30	17.9	91.1	1,497	8.8
	≥35	14.6	92.6	1,083	6.4
	≥40	8.0	96.5	541	3.2
High	≥45	4.7	98.4	280	1.6
	≥50	3.0	99.6	107	0.6
	≥55	1.9	100.0	50	0.3
	≥60	1.0	100.0	30	0.2

## Discussion

There is emerging international interest in the development of tools to flag practitioners at risk of complaints to regulators. Much of the focus has been on developing tools for physicians, for example, the Patient at Risk Score (PARS) by Hickson et al. (15). and the PRONE and PRONE-HP scores that we developed (1, 2). Apart from PRONE-HP, there have been no efforts to develop a user-friendly risk score for pharmacists. Indeed, one of the limitations of PRONE-HP is that it was developed on a sample of 14 health professions, and thus was not well tuned to the clinical context that pharmacists operate in.

This is the gap we attempted to fill in this study. Using 11 years of data from a large pharmacist regulatory body in Ontario, Canada, we showed that a risk score, which we call PRONE-Pharm, could discriminate between different levels of risk of complaints to the regulator. PRONE-Pharm uses demographic data on sex, age and country of training (which numerous studies have shown all to be related to medico-legal risk) and complaint level information on number of prior complaints and the nature of those complaints. Most of the points are assigned to the complaint information, and high scores are indicative of a higher level of complaint risk.

Decisions regarding where the line should be drawn to designate medium and high-risk pharmacists are not straight-forward and depend partly on the properties of the instrument itself (the sensitivity and specificity), the number of pharmacists classified at each level, and how the tool is to be used in practice. In terms of the trade-off between sensitivity and specificity, our starting point was that it was better to maximise specificity for medium and high-risk classifications. This is because a test with high specificity will have a low proportion of false positives. Thus, if a pharmacist scores above the threshold then it is likely they will have another complaint. (However the low sensitivity means if they score below the threshold it is unclear whether this is because they are at low risk or because of the high proportion of false negatives (16, 17)) Regarding the size of the group captured in the medium and high-risk classifications, a tool that classifies a very large number of people in either of these categories may not be useful if there are insufficient resources to offer interventions for remediation. A tool that classifies 10–15% of pharmacists as medium or high risk and 1–3% of pharmacists as high risk seemed feasible from that perspective. Finally, the effectiveness, intrusiveness and cost of any interventions that are deployed as a result of risk classification influence where the thresholds should be drawn. The exact nature of such interventions is beyond the scope of this study, but the cost of a false positive classification (for instance, undertaking a deeper file review of past complaints against a medium-risk pharmacist to look for concerning patterns of behaviour or allocating complaints against high-risk pharmacists to specialist teams for investigation) outweighs a false negative classification from a practitioner perspective. Thresholds that maximise specificity meet this goal.

These considerations led us to suggest a threshold of ≥25 for classifying medium risk practitioners. The specificity is 87% at this level (therefore only 13% of pharmacists classified as medium risk will be false positives) and around 13% of pharmacists would have a score greater than this. A score of ≥25 cannot be achieved by demographic characteristics alone—the high-risk predictors of being male, aged 50–59 years and trained internationally would only net 21 points—it would take at least one prior complaint to push a pharmacist with this profile into the medium risk category. Thus, this seems a reasonable threshold for relatively low-cost or non-intrusive interventions such as advising the pharmacist that they are at risk of future complaints or providing them with peer mentoring.

We have identified a threshold of ≥45 for classifying pharmacists as high-risk. Specificity is very high at this threshold (98%) meaning only 2% of pharmacists classified as high risk are false positives. This, combined with the small number of pharmacists in this group (1.6%), means that high-cost or intrusive interventions may be well targeted to this group.

One interesting finding to emerge from this study is that, unlike the Australian research, it does appear to be feasible to construct a risk score for pharmacists. We see two reasons for this. First, PRONE-HP was developed on 14 practitioner groups. This means that the coefficients, and therefore the points assigned to each predictor (e.g., demographic and complaint factors) came from a model averaged across a heterogenous group of practitioner groups (for example, doctors, nurses, psychologists, physiotherapists). It may be that the risk factors differ in their magnitude between these groups. Thus, it may be more fruitful in the future to develop profession-specific risk scores rather than a single overarching risk-score for multiple professions. More fundamentally, the success in developing a score here may be because of the higher degree of clustering of complaints within the pharmacists profession. In the Australian study there were 2,038 complaints against 30,778 pharmacists (19.9 per 1,000 PY). Here were observed 3,675 complaints against 17,038 pharmacists (26.6 per 1,000 PY or a 33.7% increase).

Our study has a number of strengths over previous efforts. Previous studies identifying risk and protective factors have largely used cross-sectional and case-series designs. We were able to follow pharmacists longitudinally for up to 11 years—far longer than the 5 years used in our Australian study. We were able to classify complaint issues into a taxonomy used previously, for instance distinguishing between medication use as a health issue (substance use), as a conduct issue (use and supply of medications) and as a performance issue (prescribing or dispensing). Finally, we were able to account for the changing level of risk over time by allowing some predictors to be time varying. Thus, we could account for the increased level of risk associated with the accumulation of complaints. This may in part explain why some of the seminal studies in this area were unable to develop “experience ratings” tools for medical liability insurers (18–21).

Our study also has a number of limitations. First, there are a number of important aspects of clinical care that we were not able to measure. Some of these are likely to have an important bearing on risk assessment. These could include patient volume, practice business type (independent, franchise), practice setting (community versus hospital pharmacy), patient mix and disciplinary history. Their exclusion means we are unable to assess their relationship with complaint risk and how the association with other variables changes as a result of their inclusion. Second, the complaint issue variables used in our analysis were based on an assessment at lodgement. New or different issues may have been uncovered during investigation. Third, our study uses lodgement of a complaint as the outcome. However, not all complaints will be associated with poor performance or wrongdoing. Finally, we treat each complaint as a separate and independent event. In some cases, a single complaint may generate multiple subsequent complaints, often because of publicity in the press. We were not able to link these complaints together.

## Conclusion

Some complaints to regulators represent isolated incidents; others are suggestive of underlying and persistent problems. Tools such as PRONE-Pharm have the potential to summarise the vast amount of information that regulators routinely gather to distinguish one type of complaint from the other. While prediction alone does not lead to quality improvement, prediction when combined with effective interventions does have the potential to improve the quality of care that pharmacists deliver; leading to direct benefits for patients.

## Data Availability

The data that support the research findings are owned by the Ontario College of Pharmacists and restrictions apply to the availability of these data which were used under license and so are not publicly available. Requests to access these datasets should be directed to informationmanagement@ocpinfo.com.
